# Target Eccentricity and Form Influences Disparity Vergence Eye Movements Responses: A Temporal and Dynamic Analysis

**DOI:** 10.16910/jemr.12.4.7

**Published:** 2019-12-03

**Authors:** Chang Yaramothu, Rajbir S. Jaswal, Tara L. Alvarez

**Affiliations:** 1 New Jersey Institute of Technology, Newark, NJ, USA

**Keywords:** Eye movement, vergence, target eccentricity, target form, target color

## Abstract

This study sought to investigate whether stimulation to the fovea or the parafovea with different color combinations influenced the temporal and dynamic features of 4° disparity vergence step responses. Twelve unique types of stimuli were displayed within a haploscope presented along the participant’s midsagittal plane. Vergence eye movement responses from fifteen naïve participants were recorded using video-based infrared eye tracking instrumentation. Latency and peak velocity from left and right eye movement responses were quantified. Results show that the type of stimulus projection (foveal versus parafoveal) significantly (*p*<0.001) influences the vergence response latency but did not impact peak velocity. Vergence responses to eccentric circles with 6° eccentricity targeting the parafovea resulted in a significantly faster response latency compared to vergence responses to a cross with 2° eccentricity stimuli targeting the fovea. Results have implications for the stimulus design of a variety of applications from virtual reality to vision therapy interventions.

## Introduction

Disparity vergence is stimulated by a visual target of interest being projected to the retina which is offset from where the fovea within each eye is currently located. Vergence eye movements are used to change gaze such that the eyes move disjunctively (inward or outward eye rotation). Technologies such as virtual reality (VR) interact with the near triad: specifically, the vergence, accommodation and pupil physiological systems [1]. The impact of VR on eye movements is only beginning to be investigated [2]. Applications in VR range from pain reduction interventions [3, 4] to surgery simulation tools [5–7] to vision therapy techniques [8, 9]. Hence, VR has enormous potential to be impactful to our society for a variety of applications. As VR becomes more prevalent in our society, more studies are needed to understand how different visual stimuli influence the temporal and dynamic properties of vergence eye movements.

Many investigations on disparity vergence use identical stimulus targets to the left and right eye that are of small target foveal eccentricity [10–12]. Others have studied targets with larger target eccentricities such as random dot stereograms that stimulate the parafovea and/ or perifovea [13]. However, a gap in knowledge is a study which records vergence responses from the same participants to compare the impact of different stimulus targets that are projected to the fovea compared to those projected to the parafovea on symmetrical disparity vergence step responses.

Another body of literature has studied differences between the left and right eye movement responses to the same symmetric target presented to both the left and right eye. Even when an identical stimulus is presented to the left and right eye on the midsagittal plane, differences are observed between the two eye movement responses. Horng, *et al.* (1998) studied asymmetries in eye movement responses to pure symmetric vergence stimuli and found that although both eyes eventually arrive at symmetrically convergent positions, the initial transient portion of the movements showed differences in amplitude and peak velocity [14]. Some differences may be attributed to the influence of saccades as observed in participants with normal binocular vision [15, 16]. Subsequent studies report that the frequency of saccades are correlated to vergence peak velocity in binocularly normal controls as well as those with the binocular dysfunction known as convergence insufficiency [17, 18]. Similarly, the slow fusional vergence system also reports asymmetrical behavior [19, 20] which is observed within the spatial direction of vergence responses [21, 22]. Prior literature supports that differences between the left and right eye movement responses may be observed and further investigation should study the left and right eye ocular motility individually and combined.

Vision therapy or orthoptic therapeutic interventions for the convergence system include the use of eccentric circles [23]. Eccentric circles are typically the same overall target eccentricity, but stimuli color may differ. For example, one eye may observe a red stimulus target while the other eye observes a green stimulus target. The color difference facilitates the identification of visual suppression. Eccentric circles, in addition to other techniques, are routinely used to improve convergence function in patients with convergence insufficiency. [23] It is unknown what the temporal and dynamic features of vergence step responses are when the eyes are presented with visual targets each of a different color to each eye.

This study seeks to investigate different visual stimulus factors specifically how target projections onto the fovea compared to projections onto the parafovea retinal region and color influence vergence eye movement responses through quantitative temporal and dynamic measurements. This knowledge is important for future visual stimuli design consideration for virtual reality applications as well as the stimulus design of therapeutic interventions that modify oculomotor motility.

## Methods

Fifteen participants (9 males) who did not have prior experience with eye movement experiments and did not know the aims of this investigation were enrolled in this study. All participants were between the ages of 18 and 35 years. All participants signed written informed consent forms approved by the New Jersey Institute of Technology (NJIT) Institution Review Board (IRB) in accordance with the Declaration of Helsinki. Exclusion criteria included a history of binocular dysfunction or head injury including concussion.

All participants were screened to ensure they had normal binocular vision. Participants had a normal near point of convergence (NPC) of less than 6 cm, normal positive and negative fusional range passing Sheard’s criteria [24], and normal local stereopsis of greater than 50 sec of arc. NPC was assessed by measuring the distance a high acuity target, was perceived as diplopic along the participant’s midline [25]. Fusional vergence was assessed with a prism bar. The prism bar was a step change in prismatic demand of 1Δ, 2Δ to 20Δ in increments of 2Δ, and 20Δ to 45Δ in increments of 5Δ (Bernell Corp., South Bend, IN, USA). A high acuity target from Gulden Ophthalmic was presented on the mid-sagittal plane of the participant 40 cm away. The examiner held the prism bar base-out to measure positive fusional range and base-in to measure negative fusional range. The examiner asked the participant to view the target as single and clear and recorded when the participant experienced blur and diplopia (break point). Stereopsis was assessed by the Randot Stereopsis Test (Bernell Corp., South Bend, IN, USA). Normal was defined as a stereopsis of less than or equal to 70 seconds of arc. All participants had normal or corrected to normal visual acuity. All these methods have been used in prior studies [17, 26–28].

Subjects were instructed to allow the visual target to become single by viewing the center of the visual target and to try not to scan or move their eyes around the target. For the eccentric circles, subjects were instructed to look towards the center of the stimulus and were given a brief training session outside of the haploscope where they observed eccentric circles used within clinical practice. These instructions to the subjects were believed to reduce the number of potential saccades observed within the data.

**Figure 1:**
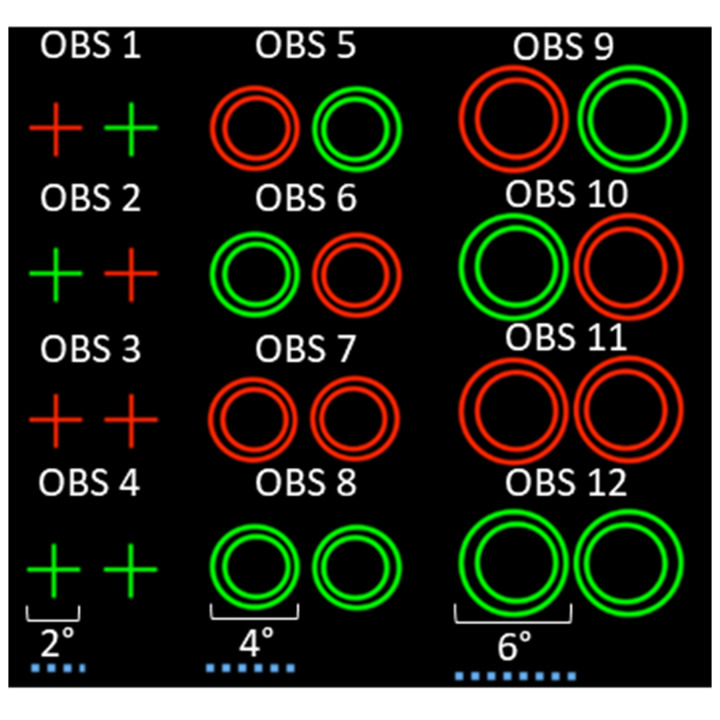
A) Stimulus design used for the 12 observation (OBS) with respect to visual target and color.

Figure 1 illustrates the stimuli targets and color combinations utilized in this study. With three different stimuli targets and four possible color pairings presented to the left and right eye, twelve different types of observations are recorded during each experimental session. The target size of the stimuli pair was chosen after carefully reviewing retinal eccentricity plots to stimulate either the fovea or parafovea. The three retinal eccentricities were 2°, 4°, and 6° in length and height to study stimuli that fall within the central fovea to the parafovea. There were 20 trials of each of the 12 observations for a total of 240 trials during the experiment. Trials were pseudo-randomly intermixed to reduce predictive cues of vergence because anticipatory cues are known to influence temporal and dynamics properties of vergence eye movement responses [29–32].

The foveal “cross” stimuli pairs used in observations 1 through 4 (Figure 1) have smaller target eccentricity (2° length by height) compared to the parafoveal eccentric circles stimuli observations 5 through 12. The stimuli were designed to be simple combination of red (R), green (G), blue (B) stimuli. The red stimulus had the RGB value of [255, 0, 0] and the green stimulus had the RGB value of [0, 255, 0]. Crosses were chosen for the smallest target eccentricity due to the preference to show the participants a high acuity target and because the eccentric circles at 2° eccentricity had poor resolution quality. The inner and outer circle were difficult to differentiate at a 2° eccentricity. While observations 5 through 8 and 9 through 12 were designed to be larger in target eccentricity of 4° and 6°, respectively their “eccentric circle” shape are used clinically in vision therapy applications [23]. The benefit of eccentric circles was it gave participants visual feedback of the inner circle appearing closer to the subject compared to the outer circle because of the displacement of the circles with respect to each other.

As determined by the retinal eccentricity plot, stimulus target eccentricity is meant to induce stimulation starting at the fovea centralis, then to parafovea and perifovea as the visual target becomes larger in target eccentricity. The fovea centralis, or central region of the retina, is populated mostly by cones [33, 34], which allow for color vision and are responsible for rendering high spatial acuity.

The color of the stimuli is also investigated across all twelve observations. Currently, there is a great deal of research involving the determination of the ratios of cone types and their arrangement throughout the retina [35–37]. As observed in a cone mosaics of the retina, the fovea is predominantly populated by medium-wavelength (green) and long-wavelength (red) cones [38]. Prior studies have investigated the human trichromatic cone mosaic in order to better understand the properties of the network of cones that allow humans to derive color across a spectrum of light [39]. Among the three cone types found in the human retina (short-wavelength [S], medium-wavelength [M], long-wavelength [L]), the M and L cones are in greater abundance than the S. As a result, the stimulus colors of red and green were chosen to stimulate the greater number of cones.

The visual stimuli were presented on a black background. The illuminance of the visual stimulus displays was measured with a Dr. Meter digital illuminance meter (Model LX1330b) which has a resolution of 0.1 lux and ranges from 0 to 200,000 lux. The black background of our visual displays had a brightness of 0.1 lux. When the entire screen was green, the maximum illumination was 1.8 lux. When the computer monitor was completely red, the maximum illumination was 0.8 lux for both the left and right monitor. Illuminance levels are important to monitor because it has been shown to impact reading speeds [40].

**Figure 2A:**
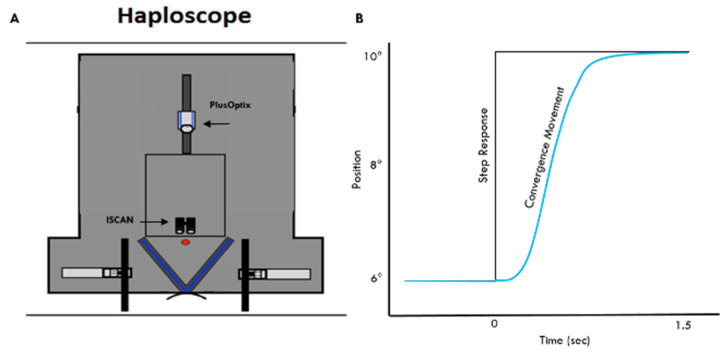
The haploscope design used to present stimuli in separate visual fields to participants. 2B: experimental protocol illustrating the presentation of stimulus and vergence eye movements induced utilizing a haploscope.

Eye movements are recorded using a custom-built haploscope (Figure 2A). The haploscope employs 50% reflective mirrors that project stimuli to the left eye and right eye individually. Symmetrical vergence stimuli are presented on the participant’s mid-sagittal plane. Hence, fusion of a single percept is attained using a vergence eye movement (Figure 2B) The ISCAN (Figure 2A) infrared tracking instrument records the induced vergence eye movement of each eye with a sampling rate of 240 frames per second for a temporal resolution of 4 msec. The haploscope allows for the study of the disparity vergence system, where stimulation of the accommodative system is held virtually constant [41].

Figure 2B illustrates a typical convergence eye movement. The experiment was composed of 4° symmetrical vergence disparity steps presented between a vergence angular demand of 6° to 10°. The participant begins with a fixation point of 6° binocular vergence angular demand and when the subject pushes a trigger push-button, the visual stimulus target is abruptly changed (step movement) to a vergence angular demand of 10°. After the first movement concludes, the participant may rest, or blink if needed before the next visual stimulus is presented. All instrumentation is controlled with a proprietary custom software suite called VisualEyes [42].

Raw data are processed and analyzed using custom MATLAB scripts. Vergence responses that contained blinks or saccades during the transient portion of response are omitted from analysis. Primary metrics used to assess left and right eye vergence eye movements are peak velocity and latency. Latency is defined as the beginning of the response movement and quantitatively identified as 5% of the total movement which for this study is a movement of 0.2° from the initial vergence angular demand. Each experimental session consists of twelve unique observations pseudo-randomized, so the same sequence of stimuli is presented to each participant. The experiment is composed of two distinct within-participant factors (stimulus type and stimulus color). As all participants mediated eye movements for each trial condition, a repeated-measures ANOVA is performed to eliminate individual differences as a source of between-group variability whereby all between-group variability would be due to differences in trial conditions (i.e. stimulus target eccentricity, color, or a combination of both). There are no individual differences between trials because all participants experience the same trial presentation order that was pseudo-randomly presented so the subject could not predict the stimulus order.

## Results

**Figure 3:**
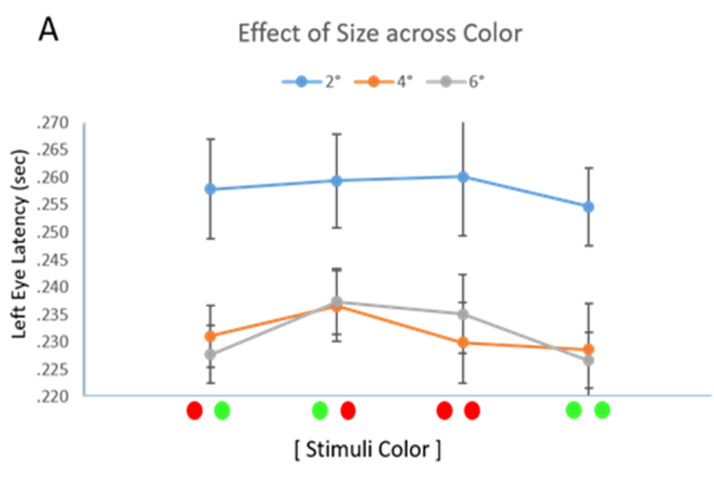

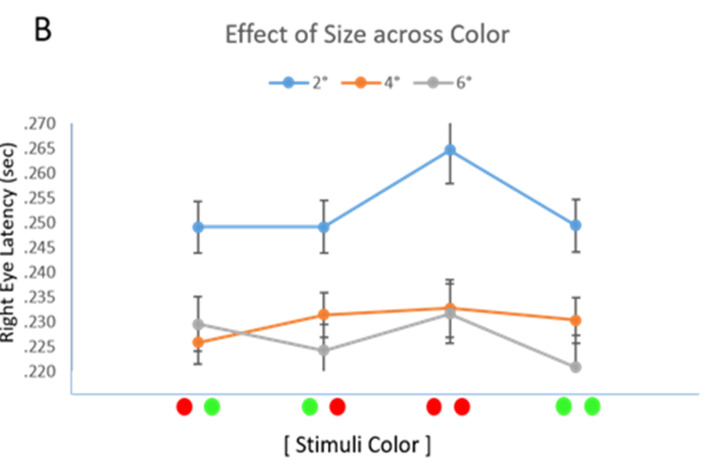
The effect of stimulus target type across color for (A) the left eye response latency and (B) right eye response latency.

Figure 3 shows the group-level analysis of the average latency with one standard deviation for the effect of stimulus type (2° cross to fovea, 4° and 6° eccentric circles to the parafovea) across all color presentations for both the left eye (3A) and the right eye (3B) latency measures. A repeated-measures ANOVA confirmed a significant effect of target type on convergence latency [F(2,28)=37.468, *p*<0.001]. The Cohen effect size was 0.89 where results of greater than 0.8 are considered a large effect size. No effect of color or the interaction between target eccentricity and color were observed with respect to response latency (*p*>0.1).

As three different target types were presented, a post-hoc paired t-test with Bonferroni correction was performed to assess potential differences between left eye and right eye latencies with respect to target type. Vergence responses of the 2° cross target projected to the fovea had significantly greater latencies than the 4° and 6° eccentric circle targets that project to the parafovea in a post hoc analysis (*p*<0.001).

**Figure 4:**
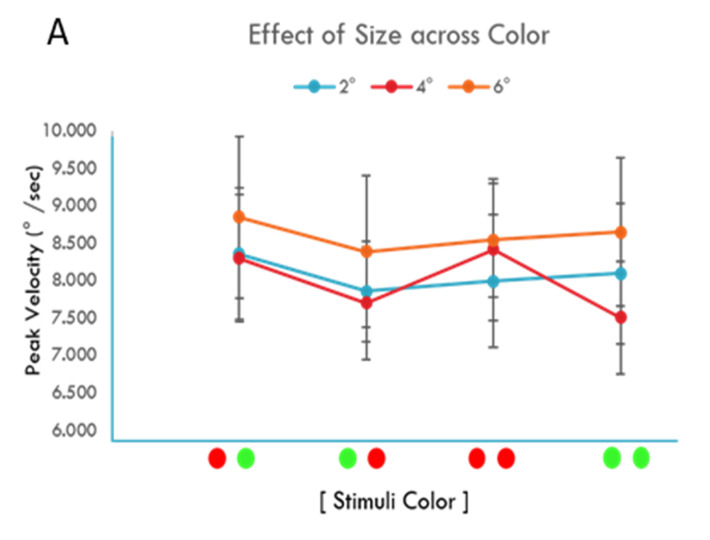

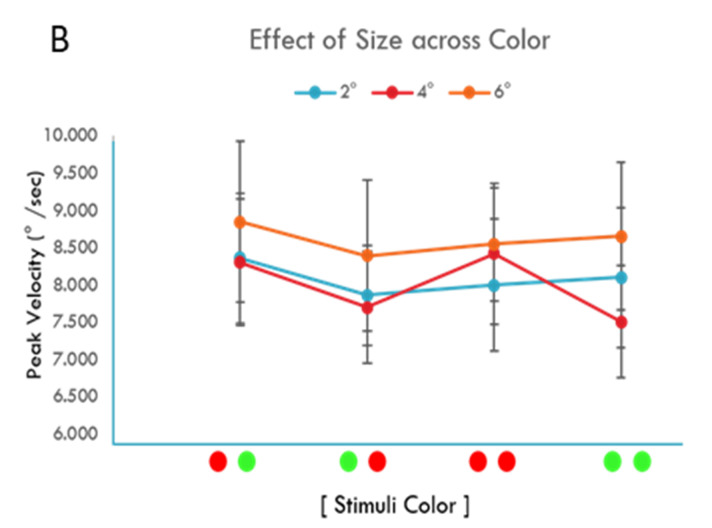
The effect of stimulus target type across color for (A) the left eye response peak velocity and (B) right eye peak response velocity.

Figure 4 is a similar nomenclature to Figure 3 except it is a group-level analysis of the average with one standard deviation of the left eye response (Figure 4A) and right eye response (Figure 4B) peak velocities. No significant effect was observed between target type, color, and their interaction with respect to left eye (Figure 4C) and right eye (Figure 4D) response peak velocities. A paired t-test of left eye and right eye average velocities across pairings of all target type groups revealed no statistically significant differences (*p* ≥ 0.28).

**Figure 5:**
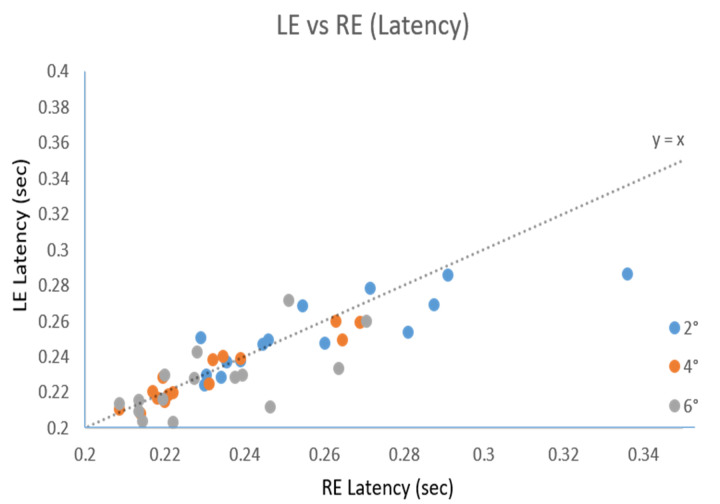
The effect of stimulus target type for the left-eye and right-eye movement response latency for the 2° foveal cross, and the 4° and 6° parafoveal eccentric circles targets stimulating 4° symmetrical vergence step movements.

Figure 5 is a scatter plot of left eye latency as a function of the right eye latency. A symmetric reference line (dotted y=x) is also provided to display how similar the latencies were between the left and right eye movement responses. If responses had identical latencies, then the data would be superimposed on this symmetry line (dotted line Figure 5). The results support that the 4° vergence step responses to the eccentric circles stimulating the parafoveal with larger target eccentricity of 6° and 4° have shorter response latencies compared to the foveal cross stimulus that had 2° target eccentricity responses. Asymmetries between the left and right eye movement responses are not observed since the results are close to the symmetry line (dotted line Figure 5).

The percentage of saccades during the transient portion of the movement defined as between the start of the movement and during the dynamic portion of the response varied based upon the eccentricity of the target. The smaller 2° eccentricity cross targets exhibited between 13% and 35% percentage of trials with saccades, 4° eccentricity concentric circle targets were between 22% and 42% and the 6° eccentricity concentric circle targets had between 31 and 55% of saccades.

## Discussion

### Stimuli Target Form Compared to Eccentricity

In this study, vergence eye movements were investigated in response to different target forms with varying eccentricities and color using the same 4° step stimulus movements. The specific targets utilized a central cross (2° eccentricity) which stimulates the fovea and eccentric circles (4° and 6° eccentricity) without central fixation which stimulates the parafovea. Results from this experiment indicate that the type of visual stimulus is an important factor when tracking a target in the visual field using disparity step vergence stimuli. The vergence responses to the eccentric circles of 4° and 6° had significantly shorter latencies compared to the cross of 2° eccentricity that stimulated the fovea. No significant difference was observed between the latency measurements from responses generated by the eccentric circles with 4° or 6° eccentricity. Hence, it cannot be concluded whether it was the visual target eccentricity (fovea compared to parafovea) or stimulus form (cross compared to eccentric circles) that led to the significant decrease in vergence response latency. What can be concluded is that the visual target either form and/or eccentricity does impact the temporal properties of disparity vergence responses. Future studies should include visual stimuli of constant shape scaled to different eccentricities. In addition, eye movement responses to different forms with the same target eccentricity should be collected. Once this future study is conducted then an analysis can be performed to determine whether it is the stimulus form or the stimulus eccentricity that has a greater influence on vergence response latency. This knowledge that the type of visual stimulus has an impact on vergence responses has direct impact for the design of vision therapy protocols and virtual reality visual stimuli.

The Convergence Insufficiency Treatment Trial (CITT) vision therapy protocol integrates a Quoit vectogram, which has a large target eccentricity (parafoveal to perifoveal stimulation) and this is an exercise introduced early on in the vision therapy protocol [43]. Clinicians typically start vision therapy protocols with larger target eccentricities and then as the therapy progresses and the patient’s vergence function improves, the target eccentricity decreases. Many past investigations report that convergence insufficiency patients have slower vergence responses compared to binocularly normal controls [8, 11, 18, 44, 45]. Future studies should investigate the impact of stimulus target form, eccentricity and color for patients with binocular dysfunctions.

The significant difference of response latency as a function of target eccentricity and form also has an impact on virtual reality, augmented reality, and mixed reality technologies. As head mounted displays become more common for entertainment, education, and therapy, careful visual stimulus design is strongly encouraged to be considered to ensure individuals can comfortably interact with the technology [46].

No statistically significant differences were observed in vergence peak velocity and latency measures with respect to the four different left eye and right eye color pairings. The target color pairings to the left eye and right eye did not significantly influence vergence-tracking dynamics. One study reported that color discrimination, even in the periphery, may be as strong as in the fovea centralis [47, 48]. Hence, while these prior studies did not study eye movements, they do conclude that color discrimination can be strong for both central and peripheral vision.

### Comparison to Other Literature

Several other studies have investigated central compared to peripheral cues. One study reports that peripheral stimuli had longer latencies compared to central stimuli although no quantitative latency values are provided [49]. Other studies report that the larger the eccentricity of the visual stimulus the more optimal the gain (output amplitude divided by input amplitude) when studying sinusoidal stimulus targets [50–52]. These investigations did not conduct a temporal analysis. Our current study did not observe a change in the dynamics of the responses as the eccentricity of the target increased but does observe a significant reduction in latency. The primary difference between these studies and ours is that our study utilizes vergence step responses compared to the prior literature that investigates vergence sinusoidal responses.

The Dual Mode Theory states that vergence can be described as a two-component system [53]. The fusion initiating component (FIC) is preprogrammed controlled and brings the eyes close to the target quickly but not necessarily very accurately [29, 54]. The fusion sustaining component (FSC) is feedback controlled and is responsible for reducing the error or the difference between where the eyes are currently located and the visual stimulus target [55, 56]. Our investigation uses step stimuli which concentrates on the FIC while the prior research that uses sinusoids is an investigation of the FSC. Neurophysiological data support that there are burst and tonic cells within the supraocular region of the midbrain which are described as velocity-encoding or preprogrammed and position-encoding or feedback controlled cells, respectively [57–59]. Since the FIC and FSC are governed by difference neural substrates, then it is possible that these cells respond to central and peripheral stimuli differently.

Another study investigated steps and ramps using central and peripheral visual stimuli where the peak velocity of central visual targets had greater velocity compared to peripheral visual targets and did not include a temporal analysis [60]. An important difference between the Hung et al. (1991) study and ours is the experience-level of the participants within oculomotor studies. Our study investigated 15 naïve subjects who did not have prior experience with any oculomotor studies and did not know the aim of our study. While Hung et al. (1991) studied 4 subjects where 3 of them were the authors who have a history of publishing vergence responses to central targets but not peripheral stimuli. Prior research shows that repetitive training on vergence eye movements increases peak velocity [61]. The peak velocity results from our naïve subjects is similar in value to the peak velocity of results from the stimuli to peripheral targets reported by Hung et al. (1991). Hung et al. (1991) has many publications using central targets only, while the 1991 investigation was their first studying peripheral targets. One potential confounding variable between our study and Hung et al.’s study is the amount of oculomotor training.

The last observation from our study is the prevalence of saccades increased as the eccentricity of the target increased. Our results are similar to other studies which analyze saccades during symmetrical vergence step responses [62, 63].

### Study Limitations

The limitations present in this current study are due to the available instrumentation for stimuli presentation. Although one of the primary objectives was to study the differences in color, in terms of various wavelengths (∼650nm for red and ∼530nm for green), a difference of illuminance was present due to the monitors used for stimuli presentation. This difference of intensity could have confounded the results. Specifically, the luminance ratio or contrast was calculated as the illuminance of the color stimulus divided by the illuminance of the background. The contrast was 8 for the red visual stimulus and 18 for the green visual stimulus. Prior research shows that for low luminance visual stimuli, the vergence system is biased towards dark vergence while medium to higher contrast stimuli results in a full vergence response [64, 65]. It is possible that the different contrast ratios masked any potential effect that color may be having on the vergence system. Future research is needed where illuminance is held constant and the color wavelength is systematically varied for the visual stimulus.

The instrumentation, additionally, limited this study to utilize a cross for the smallest of eccentricity of 2°. Thus, different stimuli forms (cross and eccentric circles) were studied. This change in form could have potentially affected the results of the study. Future studies should study the effects of eccentricity with a constant stimuli form as it is systematically changed over a large range.

In conclusion, data from this study shows that stereoscopic target color does not influence vergence peak velocity and latency of left eye or right eye movement responses. However, stimulus target type does affect both the left-eye and right-eye latency within disparity step convergence eye movements.

## Ethics and Conflict of Interest

The author(s) declare(s) that the contents of the article are in agreement with the ethics described by the Journal and that there is no conflict of interest regarding the publication of this paper.
